# Comprehensive Advanced Physicochemical Characterization and In Vitro Human Cell Culture Assessment of BMS-202: A Novel Inhibitor of Programmed Cell Death Ligand

**DOI:** 10.3390/pharmaceutics16111409

**Published:** 2024-11-01

**Authors:** Hasham Shafi, Andrea J. Lora, Haley M. Donow, Sally E. Dickinson, Georg T. Wondrak, H.-H. Sherry Chow, Clara Curiel-Lewandrowski, Heidi M. Mansour

**Affiliations:** 1 Florida International University Center for Translational Science, Port St. Lucie, FL 34987, USA; 2 University of Arizona Cancer Center, University of Arizona, Tucson, AZ 85724, USAwondrak@pharmacy.arizona.edu (G.T.W.);; 3 Department of Pharmacology, The University of Arizona College of Medicine, Tucson, AZ 85724, USA; 4 Department of Pharmacology and Toxicology, The University of Arizona College of Pharmacy, Tucson, AZ 85721, USA; 5 Division of Hematology and Oncology, Department of Medicine, The University of Arizona College of Medicine, Tucson, AZ 85724, USA; 6 Division of Dermatology, Department of Medicine, The University of Arizona College of Medicine, Tucson, AZ 85724, USA; 7 BIO5 Institute, University of Arizona, Tucson, AZ 85724, USA; 8 Department of Cellular & Molecular Medicine, Herbert Wertheim College of Medicine, Florida International University, Miami, FL 33199, USA; 9 Department of Environmental Health Sciences, Robert Stempel College of Public Health and Social Work, Florida International University, Miami, FL 33174, USA; 10 Department of Biomedical Engineering, College of Engineering and Computing, Florida International University, Miami, FL 33174, USA

**Keywords:** microscopy, phase transitions, in vitro human cell viability, transepithelial electrical resistance (TEER), molecular fingerprinting spectroscopy

## Abstract

**Background/Objectives:** BMS-202, is a potent small molecule with demonstrated antitumor activity. The study aimed to comprehensively characterize the physical and chemical properties of BMS-202 and evaluate its suitability for topical formulation, focusing on uniformity, stability and safety profiles. **Methods:** A range of analytical techniques were employed to characterize BMS-202. Scanning Electron Microscopy (SEM) was used to assess morphology, Differential Scanning Calorimetry (DSC) provided insights of thermal behavior, and Hot-Stage Microscopy (HSM) corroborated these thermal behaviors. Molecular fingerprinting was conducted using Raman spectroscopy and Fourier Transform Infrared (FTIR) spectroscopy, with chemical uniformity of the batch further validated by mapping through FTIR and Raman microscopies. The residual water content was measured using Karl Fisher Coulometric titration, and vapor sorption isotherms examined moisture uptake across varying relative humidity levels. In vitro safety assessments involved testing with skin epithelial cell lines, such as HaCaT and NHEK, and Transepithelial Electrical Resistance (TEER) to evaluate barrier integrity. **Results:** SEM revealed a distinctive needle-like morphology, while DSC indicated a sharp melting point at 110.90 ± 0.54 ℃ with a high enthalpy of 84.41 ± 0.38 J/g. HSM confirmed the crystalline-to-amorphous transition at the melting point. Raman and FTIR spectroscopy, alongside chemical imaging, confirmed chemical uniformity as well as validated the batch consistency. A residual water content of 2.76 ± 1.37 % (*w*/*w*) and minimal moisture uptake across relative humidity levels demonstrated its low hygroscopicity and suitability for topical formulations. Cytotoxicity testing showed dose-dependent reduction in skin epithelial cell viability at high concentrations (100 µM and 500 µM), with lower doses (0.1 µM to 10 µM) demonstrating acceptable safety. TEER studies indicated that BMS-202 does not disrupt the HaCaT cell barrier function. **Conclusions:** The findings from this study establish that BMS-202 has promising physicochemical and in vitro characteristics at therapeutic concentrations for topical applications, providing a foundation for future formulation development focused on skin-related cancers or localized immune modulation.

## 1. Introduction

Small-molecule inhibitors of the programmed cell death protein 1 (PD-1) and its ligand PD-L1, represent a novel non-antibody approach to cancer immunotherapy [1]. The PD-1 protein is a surface receptor present on various immune cells, including T cells, natural killer cells, and B cells, while PD-L1 is a transmembrane protein expressed or induced on both hematopoietic (e.g., immune cells such as antigen-presenting cells, T cells, and neutrophils) and non-hematopoietic cells (e.g., endothelial and epithelial cells) [2]. The interaction between PD-L1 and its surface receptor PD-1 serves as a negative regulator of immune cell activation, often referred to as an “immune checkpoint”, which plays a critical role in immune evasion by tumors [2]. Additionally, the PD-1/PD-L1 complex functions as a crucial immune checkpoint regulator, essential for maintaining immune homeostasis, preventing autoimmunity, resolving inflammatory signaling, and ensuring immune tolerance [3]. The PD-1/PD-L1 pathway is frequently exploited by tumor cells to escape immune surveillance, positioning it as a key target in cancer immunotherapy [4]. Immunotherapies have employed large-molecule monoclonal antibodies, such as pembrolizumab, atezolizumab, and nivolumab, which target the PD-1/PD-L1 interaction extracellularly [5]. In contrast, small-molecule inhibitors offer a unique mechanism of action by binding to the PD-L1 dimer interface, thereby preventing its interaction with PD-1 [6]. This binding effectively reactivates T cell-mediated immune responses against tumors, providing a distinctive therapeutic approach compared to antibody-based inhibitors. Thus, both small molecules and antibodies act to reactivate T cell-mediated immune responses against tumors through distinctive mechanisms.

**Figure 1:**
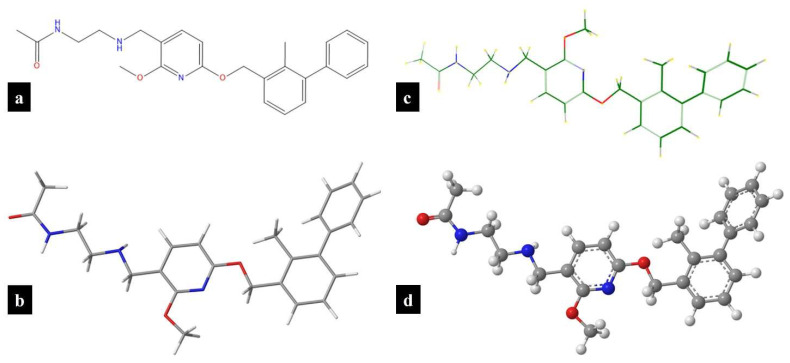
BMS-202 structures drawn using ChemDraw^®^ ver. 21.0.0 (ChemOffice, Cambridge, MA, USA): (**a**) chemical structure; and (**b**) wire-frame 3D model; and Molecular Modeling Pro Plus^®^ ver. 8.0.4 (Norgwyn Montgomery Software Inc., North Wales, PA, USA): (**c**) chemical structure; and (**d**) ball-and-stick 3D model.

The PD-1/PD-L1 axis is a hallmark of various malignancies and has been extensively documented in several tumor types, including dermatological cancers [7,8]. Blocking the interaction between PD-1 and PD-L1 has emerged as a crucial therapeutic target, particularly for treating non-melanoma skin cancers (NMSCs) such as cutaneous squamous cell carcinoma and basal cell carcinoma [8]. The incidence of skin cancers has been increasing at an alarming rate, especially in the United States over the past 20 years, primarily due to increased exposure to ultraviolet (UV) radiation from the sun [9]. Recent studies have demonstrated that PD-L1 ligands are not only overexpressed in NMSC but are also upregulated in response to environmental UV exposure [10,11]. Clinically, various systemic immunotherapies have been approved by regulatory agencies for treatment of advanced dermatological malignancies, including basal cell carcinoma, melanoma, and cutaneous squamous cell carcinoma [12,13,14]. However, the use of antibody therapies in skin cancers is limited for several reasons [15]. Antibodies are typically administered via parenteral routes, necessitating a clinical setting, which adds to the cost and inconvenience for patients [15]. Moreover, the distribution of antibodies to the tumor site is often restricted due to their large molecular size, which hinders effective skin permeation. Additionally, antibodies have a long pharmacokinetic half-life, often lasting several weeks, which can necessitate immunosuppressive treatments to manage immune-related adverse effects, thereby increasing the risk of infection [16]. The high production costs associated with antibodies, due to the need for recombinant production, and the requirement for cold chain transportation and storage further limit their use [15,17,18]. In contrast, small-molecule inhibitors of immune checkpoints address many of the limitations associated with antibody therapies [15]. Small molecules offer ease of administration, with the potential for formulation as topical or oral agents, providing greater convenience for patients. They also have a more favorable tumor distribution profile, as they can be locally applied in cases of NMSC or directed towards the tumor by optimizing their physicochemical properties [1,15]. The shorter half-life of small molecules can be advantageous in managing adverse events, allowing for more flexible dosing regimens [1,15]. Additionally, small molecules are typically less expensive to produce, and do not require special transportation or storage conditions [1,15]. These factors contribute to making small-molecule inhibitors more affordable and patient-friendly while delivering comparable clinical outcomes. Figure 1 shows the different structural representations of BMS-202, a small-molecule inhibitor of PD-1/PD-L1 axis developed by Bristol-Myers Squibb (BMS), which is the focus of the study.

BMS-202, is chemically known as *N*-(2-((2-Methoxy-6-(2-methyl-biphenyl-3-ylmethoxy)-pyridin-3-ylmethyl)-amino)-ethyl)-acetamide and is pharmacologically a small-molecule inhibitor targeting the PD-1/PD-L1 axis [19]. Structurally, BMS-202 belongs to the biphenyl scaffold derivatives and is characterized by a methoxy-1-pyridine chemical framework, which enables it to effectively bind to PD-L1, thereby blocking its interaction with PD-1 [1,19]. The antitumor, immunomodulatory, and pharmacokinetic properties of BMS-202 have been investigated in various preclinical models. For instance, Zhengping Hu et al. examined BMS-202 in B16-F10 melanoma-bearing mice, demonstrating that the molecule was well absorbed and distributed into tumors. The study revealed significant inhibition of the PD-1/PD-L1 interaction, leading to enhanced production of IFN-γ and an increase in CD8^+^ T cells, which are crucial for mounting an immune response against tumor cells [20]. Additionally, research has explored the antitumor activity of BMS-202 in glioblastoma cells, where it was found to inhibit cell proliferation, migration, and invasion by reducing PD-L1 expression [21]. Furthermore, BMS-202 has been studied as a prophylactic agent against UV-induced skin damage and potential skin cancers associated with UV-induced PD-L1 overexpression. For example, a study by SE Dickinson et al. showed that the topical application of BMS-202, dissolved in acetone, significantly antagonized UV-induced PD-L1 expression and provided protective effects against cutaneous photodamage, as shown by reduced inflammatory reactions and reduced epidermal apoptosis [10]. However, while no irritation was noted in the above studies using BMS-202, previous work has predominantly utilized non-pharmaceutical approaches and solvents that are not generally regarded as safe (GRAS) due to issues such as potential skin irritation and lack of clinical relevance. Given these limitations, there is a critical need to develop pharmaceutical-grade topical formulations of BMS-202, such as creams, gels, lotions, or ointments. These formulations can improve skin permeation, maintain skin hydration, enhance stability, and prevent skin irritation, thereby providing a more clinically relevant and patient-friendly approach for the topical application of BMS-202.

This study aims to provide a comprehensive evaluation of BMS-202 for potential topical applications by characterizing its physicochemical properties, stability, and safety profile on skin epithelial cells. Scanning Electron Microscopy (SEM) is employed to determine the surface morphology, while DSC and Hot-Stage Microscopy (HSM) are used to assess thermal properties and crystallinity. Raman and Fourier Transform Infrared (FTIR) spectroscopy, alongside chemical imaging techniques, are utilized to evaluate chemical uniformity and crystallinity, ensuring the consistency of the active pharmaceutical ingredient (API). Karl Fischer Titration (KFT) and Gravimetric Vapor Sorption (GVS) analyses are conducted to determine moisture content and hygroscopicity, assessing stability under varying humidity conditions. Furthermore, the cytotoxic effects of BMS-202 are evaluated using two different skin epithelial cell lines, HaCaT and NHEK, to understand its potential toxicity and impact on skin barrier integrity, as measured by Transepithelial Electrical Resistance (TEER) assays. These evaluations are critical for optimizing topical formulations to ensure efficacy and safety in clinical use.

## 2. Materials and Methods

### 2.1. Materials

BMS-202, [*N*-(2-((2-Methoxy-6-(2-methyl-biphenyl-3-ylmethoxy)-pyridin-3-ylmethyl)-amino)-ethyl)-acetamide; C_25_H_29_N_3_O_3_; molecular weight: 419.52 g/mol; purity: 98.0%] was procured from Ambeed, Inc. (Arlington Heights, IL, USA). Methanol (HPLC grade) was obtained from Fisher Scientific (Fair Lawn, NJ, USA). Ultra-high purity (UHP) nitrogen gas was supplied by Airgas (Randor, PA, USA). Hydranal™-Coulomat AD reagent was acquired from Honeywell Fluka™ (St. Louis, MO, USA), while anhydrous methanol for Karl Fischer titration was sourced from Millipore Corporation (Billerica, MA, USA). Ultra-pure water, generated using a Milli-Q P-QOD setup (Millipore, Fair Lawn, NJ, USA), was employed throughout the study.

HaCaT immortalized human keratinocytes were procured from AddexBio^®^ (T0020001, San Diego, CA, USA). Primary normal human epidermal keratinocytes (NHEKs) and KGM™ Gold Keratinocyte Growth Medium BulletKit™ were sourced from Lonza (Walkersville, MD, USA). Collagen I (rat tail), Advanced Dulbecco’s Modified Eagle’s Medium (ADMEM), and Fetal Bovine Serum (FBS) were purchased from Gibco^®^. Cell culture essentials, including penicillin–streptomycin (10,000 U/mL), amphotericin B (Fungizone), 96-well black/clear bottom plates, and Falcon™ tissue culture T75 flasks, were obtained from Thermo Fisher Scientific™ (Miami, FL, USA). Transwell^®^ Costar plates (12-well, 12 mm inserts, 0.4 µm polyester membrane) were purchased from Corning^®^, Fisher Scientific (Suwanee, GA, USA). Resazurin sodium salt was acquired from Acros Organics (Thermo Fisher Scientific Inc., Fairlawn, NJ, USA), and Dimethyl Sulfoxide (DMSO) was sourced from Millipore-Sigma (St. Louis, MO, USA).

### 2.2. Methods

#### 2.2.1. Predictive In Silico Modeling of Physicochemical Properties

The physicochemical properties of BMS-202 were computationally predicted using integrated software and quantitative structure-property models. ChemDraw^®^ ver. 21.0.0 (ChemOffice, Cambridge, MA, USA), Chem3D^®^ ver. 21.0.0 (ChemOffice, Cambridge, MA, USA), and Molecular Modeling Pro Plus^®^ ver. 8.0.4 (Norgwyn Montgomery Software Inc., North Wales, PA, USA) were employed for this purpose. Computational models were constructed to predict various physicochemical properties of BMS-202, including molecular formula, exact mass, molecular weight, octanol–water partition coefficient (LogP), calculated LogP (CLogP), total polar surface area (tPSA), water solubility (LogS), pKa, and melting point (MP).

#### 2.2.2. Scanning Electron Microscopy (SEM)

The surface morphology of the raw BMS-202 powder was examined using a ThermoFisher Phenom ProX G6 scanning electron microscope (NanoScience Instruments, ThermoScientific Instruments, Phoenix, AZ, USA), following methods previously reported [22,23,24,25]. Powder specimens were carefully affixed onto aluminum stubs using double-sided adhesive carbon tabs (Ted Pella, Inc., Redding, CA, USA). A platinum alloy coating, approximately 7 nm thick, was uniformly applied using a Luxor Platinum sputter coater (NanoScience Instruments, Phoenix, AZ, USA) under argon plasma conditions (Airgas, Air Liquide, FL, USA). SEM micrographs were captured using a Secondary Electron Detector (SED) at various magnifications, operating at an accelerating voltage of 10 kV, with a working distance of approximately 7–8 mm. The intensity was adjusted for imaging using the Phenom ProX G6 software, v6.8.1 (NanoScience Instruments, Phoenix, AZ, USA).

#### 2.2.3. Energy-Dispersive X-Ray (EDX) Spectroscopy

Elemental fingerprinting and impurity detection in the raw BMS-202 powder were performed using energy-dispersive X-ray (EDX) spectroscopy with a ThermoFisher Phenom ProX G6 system (NanoScience Instruments, Phoenix, AZ, USA) at an accelerating voltage of 15 kV according to previously published data [26]. Spot size adjustments were made to achieve a dead time of 20–30 s.

#### 2.2.4. Differential Scanning Calorimetry (DSC)

The thermal properties and phase transitions of the raw BMS-202 powder during heating were assessed using a TA Discovery DSC250 system (TA Instruments, New Castle, DE, USA) equipped with T-Zero^®^ technology and an automated computer-controlled RSC-90 cooling system, following established protocols [22,23,24,27,28]. Samples weighing between 2 and 4 mg were placed into hermetic anodized aluminum T-Zero^®^ DSC pans and sealed with aluminum lids using a T-Zero hermetic press (TA Instruments, New Castle, DE, USA). An empty hermetically sealed T-Zero^®^ aluminum pan served as the reference. The samples were heated from an initial temperature of 0.00 °C to a final temperature of 400.00 °C at a rate of 5.00 °C/min, with ultra-high purity (UHP) nitrogen purging at a flow rate of 50 mL/min. All experiments were conducted in triplicate, and data analysis was performed using TRIOS software (v5.5.0.323).

#### 2.2.5. Hot-Stage Microscopy (HSM) Under Cross-Polarizers

The phase transitions of raw BMS-202 powders were observed using hot-stage microscopy (HSM) under cross-polarizers. A Leica polarized microscope (Leica DMLP, Wetzlar, Germany) equipped with a Mettler FP82 hot stage (Mettler-Toledo, LLC, OH, USA) was employed, following previously published protocols [22,23,24]. A γ530 nm U-TP530 filter (Olympus, PA, USA) was used to filter the light. The powders were placed on glass slides and heated from 20.0 °C to 300.0 °C at a rate of 5.0 °C/min using the Mettler FP82 hot stage, which was connected to a Mettler FP 80 central processor heating unit. Digital images were captured with a Nikon Digital Sight 1000 camera featuring 10× optical zoom, controlled by Nikon’s NIS Elements software, v5.21.00 (Nikon Coolpix 8800, Nikon, Tokyo, Japan).

#### 2.2.6. Raman Spectroscopy

Raman spectroscopy molecular fingerprinting of the raw BMS-202 powder was conducted using a DXR™ Raman system (Thermo Scientific™, Fitchburg, WI, USA) in accordance with established procedures [23,24,28,29]. The Raman spectra were acquired using a 785 nm diode laser set at an intensity of 30 mW, with each spectrum derived from 16 sample exposures of 4 s each. Data acquisition involved a confocal hole of 50 µm and a grating of 400 lines/mm. Prior to analysis, baseline correction and Raman intensity smoothing were applied to the spectra, with the final spectrum representing the average of three recorded spectra. Data analysis was performed using OMNIC™ for Dispersive Raman v9.12.1019 and OMNIC Atlµs™ v9.12.990 software.

#### 2.2.7. Confocal Raman Microscopy (CRM)

Confocal Raman microscopy (CRM) was employed to generate detailed Raman imaging maps of raw BMS-202 powders using a DXR™ Raman system (Madison, WI, Thermo Scientific™, Fitchburg, WI, USA) integrated with an Olympus BX41 confocal optical microscope equipped with bright-field illumination (Olympus America, Inc., Chester Valley, PA, USA) using published protocols [24,26]. A 10× objective was used, with precise 10 µm steps along the X-Y axes to create a grid of 9 individual Raman acquisitions. Each point in this grid underwent 16 sample exposures, with the detector capturing data for 4 s per exposure. Baseline correction procedures were meticulously applied prior to analysis to ensure the accuracy of the acquired spectra. The resulting Raman maps were analyzed using OMNIC™ for Dispersive Raman v9.12.1019 and OMNIC Atlµs™ v9.12.990 software, enabling a detailed examination of the spatial distribution of Raman-active species within the BMS-202 sample.

#### 2.2.8. Attenuated Total Reflectance–Fourier Transform Infrared (ATR-FTIR) Spectroscopy

The ATR-FTIR spectra for molecular fingerprinting of the raw BMS-202 powder were recorded using a Nicolet™ iS50 FTIR Spectrometer (ThermoScientific™, Madison, WI, USA) equipped with a deuterated triglycine sulfate (DTGS) detector. Each spectrum was acquired over 32 scans with a spectral resolution of 8 cm^−1^, covering the range of 4000–700 cm^−1^, and recorded in triplicate for accuracy. A background spectrum was also collected under identical conditions. Data analysis was performed using OMNIC v9.12.928 and OMNIC Atlµs™ v9.12.990 software, with subsequent baseline correction and smoothing applied. Similar experimental conditions have been reported previously by our group [24,30,31].

#### 2.2.9. Fourier Transform Infrared (FTIR) Microscopy

FTIR microscopy was performed using a Nicolet™ Continuum™ Infrared Microscope (ThermoScientific™, Madison, WI, USA), equipped with a mercury–cadmium–telluride (MCT/A) detector according to a previously published protocol [32]. The raw BMS-202 powder was mapped for molecular fingerprinting using a 15× objective, with 10 µm steps along the X and Y dimensions, resulting in a total of 9 acquisitions. Each spectrum was collected over 32 scans within the range of 4000–700 cm^−1^. Prior to analysis, background subtraction, baseline correction, and smoothing were applied using OMNIC software (v9.12.928 and v9.12.990).

#### 2.2.10. Karl Fisher (KF) Coulometric Titration

The residual water content in the BMS-202 powder was determined using Karl Fischer (KF) coulometric titration, in accordance with established laboratory protocols [23,24,28,33]. The titration was performed with a TitroLine^®^ 7500 KF trace titrator (SI Analytics, Weilheim, Germany). Approximately 2–3 mg of powder was accurately weighed and introduced into a reaction cell containing Hydranal^®^ Coulomat AD reagent (Honeywell Fluka™, Seelze, Germany). The residual water content was calculated as the average of three independent measurements.

#### 2.2.11. Gravimetric Vapor Sorption (GVS)

Water vapor sorption isotherms were determined gravimetrically using a Discovery SA-Dynamic Vapor Sorption Analyzer (Waters™, TA Instruments, New Castle, DE, USA), following a protocol reported elsewhere [34]. The analyzer is equipped with a symmetrical microbalance and an autosampler. Raw BMS-202 powders, weighing between 1.0 and 1.5 mg, were initially dried at 25 °C and 0.0% relative humidity (RH) for up to 3 h, with the equilibrium criterion set to a weight change of ≤0.0001% within a 10-min interval. Subsequently, samples were subjected to an automated sequence where RH was incremented by 5%, ranging from 0.0% to 95% RH, with each step lasting 3 h. Equilibrium was defined as a weight change of ≤0.03% (*w*/*w*) within a 10-min interval.

#### 2.2.12. Dose-Dependent In Vitro Cell Viability on Skin Epithelial Cells

Cell viability was assessed using the Resazurin Assay to evaluate the effects of dose-dependent concentrations of raw BMS-202 powders on HaCaT (Passage number 10) and NHEK (Passage number 7) cells, following previously established protocols [29,30,31,35,36]. HaCaT cells were cultured in collagen-coated T-75 flasks with Advanced Dulbecco’s Modified Eagle’s Medium (ADMEM) supplemented with 10% (*v/v*) fetal bovine serum (FBS), Fungizone (0.5 µg/mL amphotericin B and 0.41 µg/mL sodium deoxycholate), 2 mM L-glutamine, and penicillin–streptomycin (100 units/mL penicillin and 100 µg/mL streptomycin) in a humidified incubator at 37 °C with 5% CO_2_. NHEK cells were cultured in collagen-coated T-75 flasks with KGM™ Gold™ Keratinocyte Growth Medium BulletKit™, prepared by adding KGM™ Gold™ SingleQuots™ supplements to KBM™ Gold™ Basal Medium (non-supplemented), according to the manufacturer’s instructions, in a humidified incubator at 37 °C with 5% CO_2_.

For viability assessment, cells were harvested upon reaching approximately 80% confluency using trypsin–EDTA solution at 37 °C and seeded in 96-well black plates at a concentration of 5000 cells/well in 100 μL per well. After 48 h of incubation for attachment and monolayer growth, cells were exposed to varying concentrations of BMS-202 powder (0.1 µM, 1 µM, 10 µM, 100 µM, and 500 µM). Solutions were prepared by dissolving BMS-202 powder in 10% dimethyl sulfoxide (DMSO) and 90% non-supplemented media (Un-supplemented ADMEM or KBM™ Gold™ Basal Medium). Each well received 100 µL of the prepared solution or control (10% DMSO and 90% un-supplemented media).

Following 48 h of exposure at 37 °C with 5% CO_2_, 100 μL of 20 µM resazurin sodium salt (prepared in non-supplemented media) was added to each well and incubated for 4 h. Fluorescence intensity of resorufin, a metabolite of the resazurin dye produced by viable cells, was measured at 544 nm (excitation) and 590 nm (emission) using the Synergy H1 Multi-Mode Reader (BioTek Instruments, Inc., Winooski, VT, USA). Relative cell viability was calculated based on the fluorescence intensity.

(1) $$Relative\;viability\;(\%)=\frac{Sample\;fluorescence\;intensity}{Control\;fluorescence\;intensity}\times 100$$

#### 2.2.13. In Vitro Transepithelial Electrical Resistance (TEER) with Skin Epithelial Cells at the Air–Liquid Interface (ALI)

TEER was measured across a monolayer of HaCaT cells to assess barrier tightness, integrity, and recovery following treatment with BMS-202 according to previously published data [25,31,37]. Cells were cultured in a growth medium consisting of ADMEM with 10% (*v/v*) FBS, penicillin–streptomycin (100 U/mL penicillin and 100 µg/mL streptomycin), and Fungizone (0.5 µg/mL amphotericin B and 0.41 µg/mL sodium deoxycholate) in a humidified incubator at 37 °C with 5% CO_2_. Upon reaching confluence, the cells were trypsinized and seeded at a concentration of 500,000 cells/well into the apical side of Costar Transwell^®^ inserts (0.4 µm polyester membrane, 12 mm for a 12-well plate), with 0.5 mL of media on the apical side and 1.5 mL on the basolateral side. Media was refreshed every other day from the basolateral side.

After approximately one week, when the cells formed a confluent monolayer visible under the microscope, TEER values were measured using an STX4 open-ended electrode connected to the EVOM™ Manual TEER Measurement Meter (World Precision Instruments, Sarasota, FL, USA). Under liquid-covered culture (LCC) conditions, when TEER values stabilized (7 days post-seeding) at around 170 Ω·cm², media from the apical side was removed to establish air–liquid interface (ALI) conditions for 72 h. Once the TEER values stabilized around 160 Ω·cm² under ALI conditions, the cells were exposed to 100 µM BMS-202 solution, prepared in a 9:1 ratio of non-supplemented ADMEM and dimethyl sulfoxide (DMSO). TEER values were recorded 3 h post-exposure, with subsequent measurements taken every 24 h for up to 7 days using the STX4 open-ended electrode. Simultaneous TEER recordings were made for naïve (non-treated) and vehicle (9:1 solution of non-supplemented ADMEM and DMSO)-treated cells in the Transwells^®^. TEER measurements were recorded after removing 1.5 mL of media from the basolateral side, followed by the addition of 0.5 mL of PBS (pH 7.4) to the apical side and 1.5 mL of PBS to the basolateral side of the Transwells^®^. After recording, cells were returned to ALI conditions with 1.5 mL of media on the basolateral side. Data were analyzed and plotted using GraphPad Prism^®^ v10.2.3 (GraphPad Software, Inc., Boston, MA, USA), with kinetics of TEER values presented as the percentage response of the control (TEER values at t = 0 h) using Equation (2).

(2) $$TEER\left(\%Control\right)=\frac{Sample\;TEER\;value}{Control\;TEER\;value}\times 100$$

## 3. Results

### 3.1. Predictive In Silico Modeling of Physicochemical Properties

The computational values of various physiochemical properties of BMS-202 predicted by different software are summarized in Table 1. The results indicate that BMS-202 has a molecular weight of 419.48 g/mol, which is suitable for passive diffusion through the skin barrier, although smaller molecules are generally preferred. The logP values, ranging from 4.37 to 5.10, indicate significant lipophilicity, which is favorable for skin permeability, as lipophilic molecules tend to penetrate the lipid-rich stratum corneum more effectively [38]. The pKa value of 8.08 suggests that BMS-202 will largely remain un-ionized at physiological pH, facilitating its absorption through the skin layers. The topological polar surface area (tPSA) of 75.64 Å², which is less than 140 Å², supports predictions of good skin penetration [38].

### 3.2. Scanning Electron Microscopy (SEM)

**Figure 2:**
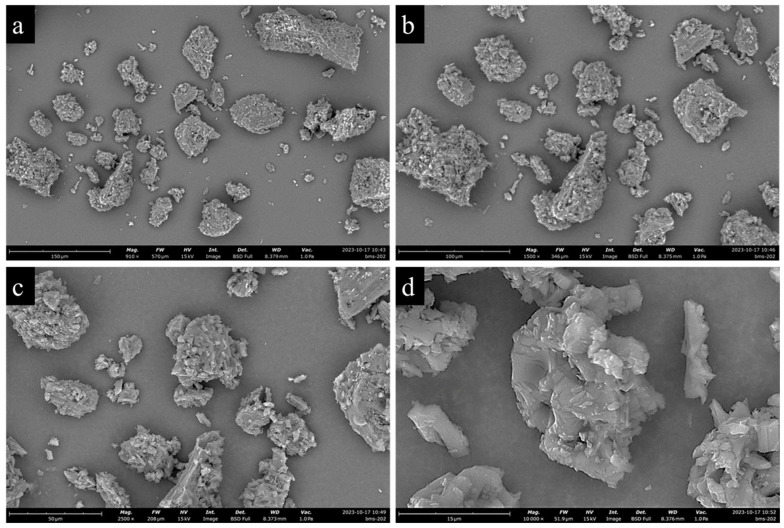
Representative SEM images of BMS-202 showing powder morphology at different magnifications: (**a**) 910×, (**b**) 1500×, (**c**) 2500×, and (**d**) 10,000×.

Figure 2 shows SEM micrographs of BMS-202 powder particles at different magnifications, revealing agglomerated particles with irregular shapes and rough surfaces, formed through the interlocking of tabular, crystal-like structures. The particles have pores and cracks throughout, and the agglomeration is composed of clusters of micro-crystallites forming a hollow structure. Particle size analysis conducted using ImageJ, v2.9.0 (U.S. National Institutes of Health, Bethesda, MD, USA) indicates a wide size distribution, with an average geometric diameter of 30.91 ± 19.43 µm and a range of 8.31–92.61 µm.

### 3.3. Energy-Dispersive X-Ray (EDX) Spectroscopy

**Figure 3:**
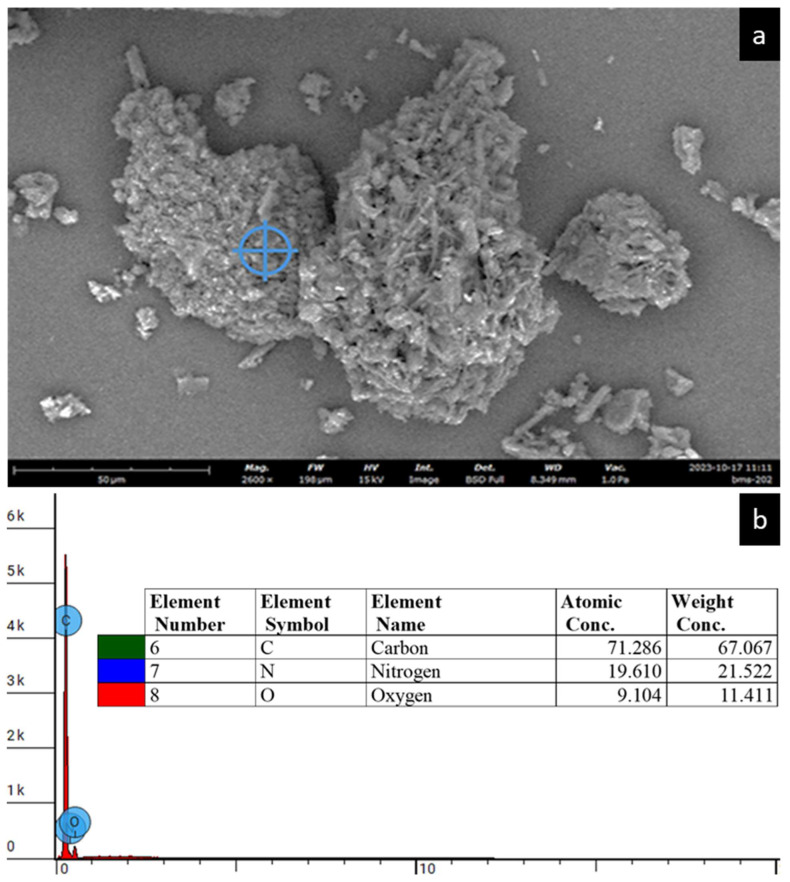
(**a**) Representative SEM micrograph taken at 2400× magnification, depicting the elemental spot analysis of the BMS-202 particle surface using EDX spectroscopy. (**b**) Representative EDX spectrum of the analyzed spot on the powder sample, indicated by the blue crosshair, illustrating the elemental composition of the BMS-202 particle, comprising C, N and O (with the N symbol overlapped by the O symbol). The inset table presents the atomic and weight concentrations of the identified elements.

The EDX spectra of BMS-202 as shown in Figure 3 reveal its elemental fingerprint, with carbon (C), nitrogen (N), and oxygen (O) constituting the primary elements of the compound′s molecular structure. The observed spectrum aligns with the manufacturer′s stated purity of 98%, and no trace impurities, particularly heavy elements, were detected.

### 3.4. Differential Scanning Calorimetry (DSC)

**Figure 4:**
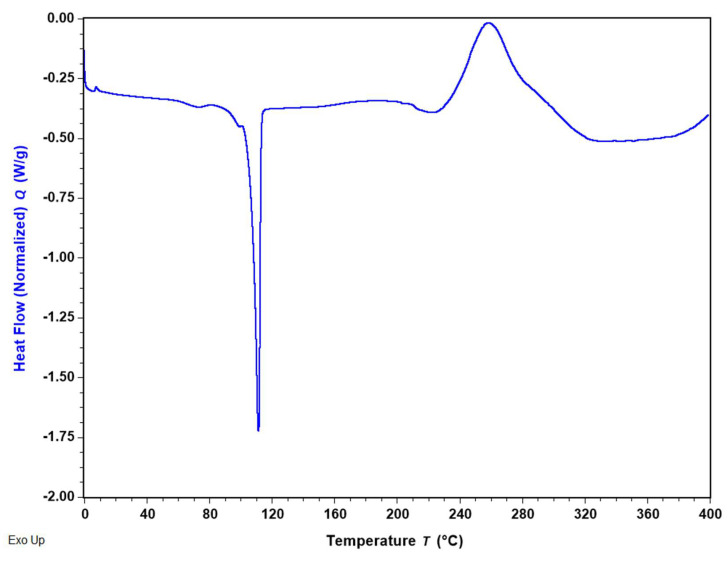
Representative DSC thermogram of BMS-202 showing main thermal transitions of the compound.

Figure 4 displays the DSC thermogram of BMS-202 powder, illustrating the primary thermodynamic transitions of the molecule. The detailed parameters for these transitions, calculated using TRIOS software (v5.5.0.323), are summarized in Table 2**.** The thermal analysis of BMS-202 up to 400 °C revealed both endothermic and exothermic transitions. The sharp endothermic peak at 110.90 ± 0.54 °C corresponds to the melting point of the compound, indicating a phase transition from solid to liquid. This transition, which requires a heat energy of 84.41 ± 0.38 J/g, suggests strong intermolecular forces in the crystalline solid state. The compound remains stable in the liquid phase up to 240 °C, after which an exothermic transition occurs. The exothermic peak, centered at 260.87 ± 2.02 °C, is broad with a high enthalpy change of 198.92 ± 9.85 J/g, suggesting significant energy release, likely due to thermal decomposition. The presence of a high-temperature exothermic peak indicates that BMS-202 maintains stability up to 240 °C before undergoing substantial degradation.

### 3.5. Hot-Stage Microscopy (HSM) Under Cross-Polarizers

**Figure 5:**
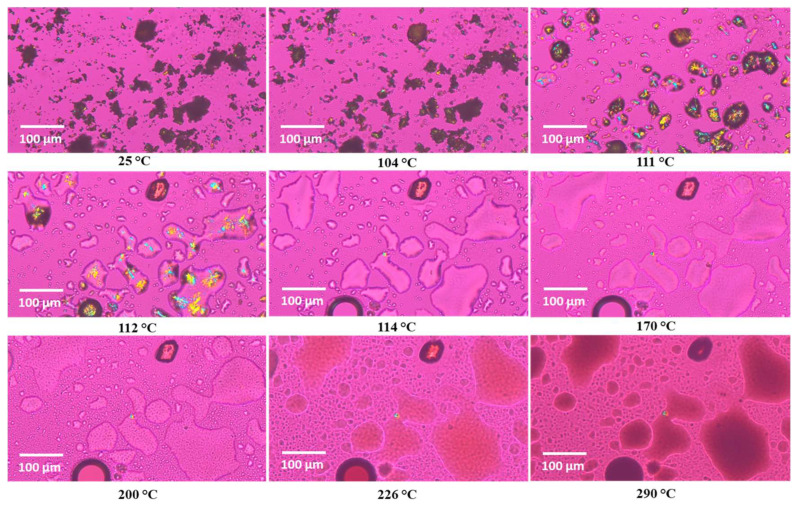
Representative HSM images of BMS-202 powder taken at different temperatures (scale 100 µm). The interference colors, particularly as the powder begins to melt, indicate birefringence.

Figure 5 shows HSM images of BMS-202 powder at various temperatures. Below and around the melting point (110.90 ± 0.54 °C), as identified in the DSC experiment, the powder demonstrates birefringence, resulting in colored pattern in the crystals. These observations confirm that BMS-202 is crystalline in nature [39]. Upon reaching the melting point, the material transitions into an amorphous glassy state, losing its ordered structure and birefringence. The HSM results support the DSC data, confirming the crystalline nature of BMS-202 and providing consistent melting point information.

### 3.6. Raman Spectroscopy

**Figure 6:**
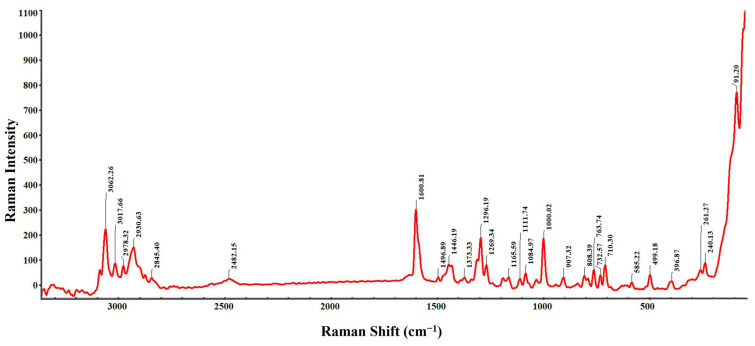
Representative Raman spectrum of BMS-202 powder showing characteristic Raman vibrational modes.

The Raman spectrum of BMS-202, shown in Figure 6, displays sharp and distinct peaks, characteristic of a crystalline structure. The sharpness of these peaks indicates that the molecules are well ordered, allowing for clear identification of distinct vibrational modes. Each peak corresponds to a specific vibrational mode of the molecule. The peaks observed at 3062.26, 3017.66, 2978.32, 2930.63, 2845.40, and 2482.15 cm^−1^ are attributed to C-H stretching in both aromatic and aliphatic chains [40]. The variety of peaks for C-H stretching arises due to different chemical environments surrounding the C-H bonds. Similarly, Raman shifts at 1446.19, 1373.33, 1000.02, 907.32, 808.39, 763.74, 732.57, and 710.30 cm^−1^ correspond to C-H bending vibrations in aromatic and aliphatic settings [40]. Distinct peaks at 1600.81 cm^−1^ and 1496.89 cm^−1^ are associated with the stretching vibrations of C=N and C=C bonds within the aromatic ring (pyridine), respectively [40]. Peaks at 1269.19 cm^−1^ and 1269.34 cm^−1^ likely represent C-N stretching vibrations from amine groups attached to the pyridine ring. C-O stretching vibrations, due to methoxy groups on pyridine or biphenyl rings, are observed at 1165.59, 1111.74, and 1084.97 cm^−1^, indicating different chemical environments [40]. The Raman shifts at 585.22, 396.87, 261.27, 240.13, and 199.18 cm^−1^ correspond to C-C stretching vibrations in aromatic rings and aliphatic chains [40]. Additionally, a low-frequency vibrational mode at 91.20 cm^−1^ is indicative of lattice vibrations, further emphasizing the crystalline nature of BMS-202 powder [41].

### 3.7. Raman Mapping

**Figure 7:**
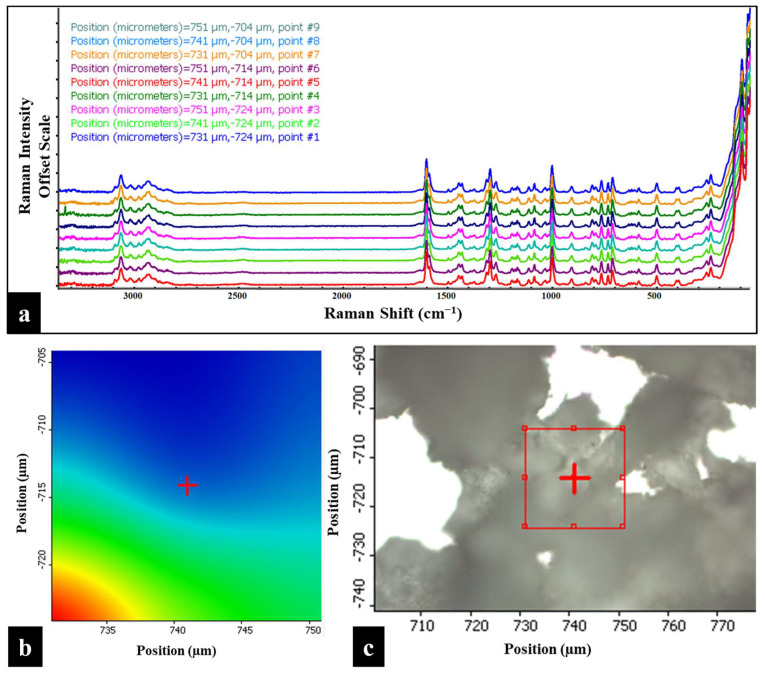
(**a**) Raman map of BMS-202 powders showing spectra obtained from nine distinct points on the sample. The Y-axis of each overlay map is presented on an offset scale for clarity. (**b**) 2D Raman area contour map of the highlighted red square region (as shown in (**c**)), indicating the position coordinates of each spectrum. (**c**) 2D Raman area map depicting the sample surface under a 10× objective, with a red square highlighting the specific region analyzed. Each spectrum in the overlay corresponds to one of the nine points within the highlighted red square, captured using a 785 nm laser, and is color-coded according to the spectrum obtained based on position coordinates.

The Raman chemical imaging map of BMS-202 powder, created by analyzing the Raman spectra at nine distinct points (position coordinates), is shown in Figure 7. This map provides a detailed view of the spatial distribution and crystallinity of the powder. The spectra overlay consistently across different regions without any deflections, indicating uniformity within the sample. The spatial distribution also demonstrates the homogeneity of the powder bed and confirms the absence of significant impurities, which is crucial for maintaining the consistency of the active pharmaceutical ingredient. The sharp and distinct peaks observed at each position coordinate, further indicating a well-defined crystalline structure in the powder sample [42]. These findings are consistent with the results from the DSC and HSM studies.

### 3.8. Attenuated Total Reflectance–Fourier Transform Infrared (ATR-FTIR) Spectroscopy

**Figure 8:**
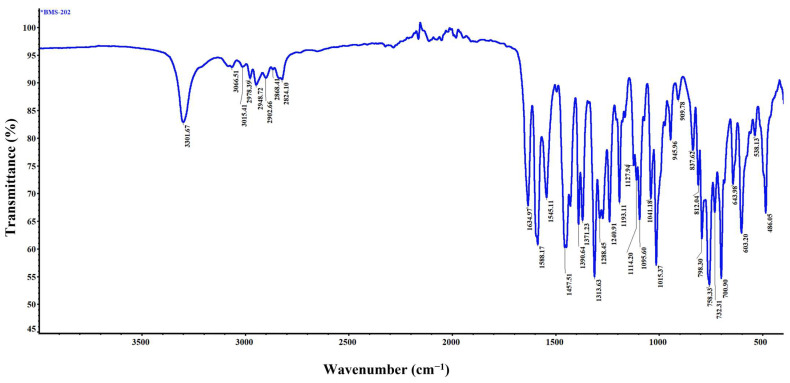
Representative ATR-FTIR spectrum illustrating the characteristic molecular fingerprints of BMS-202 powder.

The representative ATR-FTIR spectrum of BMS-202, as shown in Figure 8, provides detailed insights into the functional groups present in the molecule and aids in its identification. The peak at 3301.67 cm^−1^ corresponds to the N-H stretching, indicative of both amine and amide functionalities within the compound [43,44]. Peaks observed at 3066.51 cm^−1^ and 3051.41 cm^−1^ are associated with the C-H stretching vibrations of aromatic rings, while the peaks at 2978.39 cm^−1^, 2948.72 cm^−1^, 2902.66 cm^−1^, 2868.41 cm^−1^, and 2824.10 cm^−1^ correspond to the C-H stretching vibrations in alkyl chains [43,44]. A prominent peak at 1634.97 cm^−1^ represents the C=O stretching vibration of the amide group [44]. The aromatic C=C stretching vibrations, specifically within pyridine and phenyl rings, are reflected by peaks at 1588.17 cm^−1^ and 1545.11 cm^−1^ [44]. Peaks at 1313.63 cm^−1^ and 1288.45 cm^−1^ correspond to C-N stretching vibrations, indicative of amine and amide groups [44]. Additionally, CH_3_ bending vibrations in the alkyl chains are observed at 1390.64 cm^−1^ and 1371.23 cm^−1^, while CH_2_ bending is represented at 1457.51 cm^−1^ [44]. The ether linkage (C-O-C/C-O) vibrations are notably prominent and widespread, reflecting a diverse chemical environment surrounding both aromatic and aliphatic groups. These are observed at 1240.91 cm^−1^, 1193.11 cm^−1^, 1127.94 cm^−1^, 1114.20 cm^−1^, 1095.61 cm^−1^, 1041.18 cm^−1^, and 1015.37 cm^−1^ [44]. The aromatic C-H bending vibrations are attributed to peaks at 945.96 cm^−1^, 909.78 cm^−1^, 837.62 cm^−1^, 812.04 cm^−1^, and 798.30 cm^−1^, while bending C-H vibrations in the alkyl groups are observed at 723.31 cm^−1^ and 700.91 cm^−1^ [44]. This spectrum clearly identifies the various functional groups present in BMS-202, corroborating its structural and molecular characteristics.

### 3.9. FTIR Microscopy

**Figure 9:**
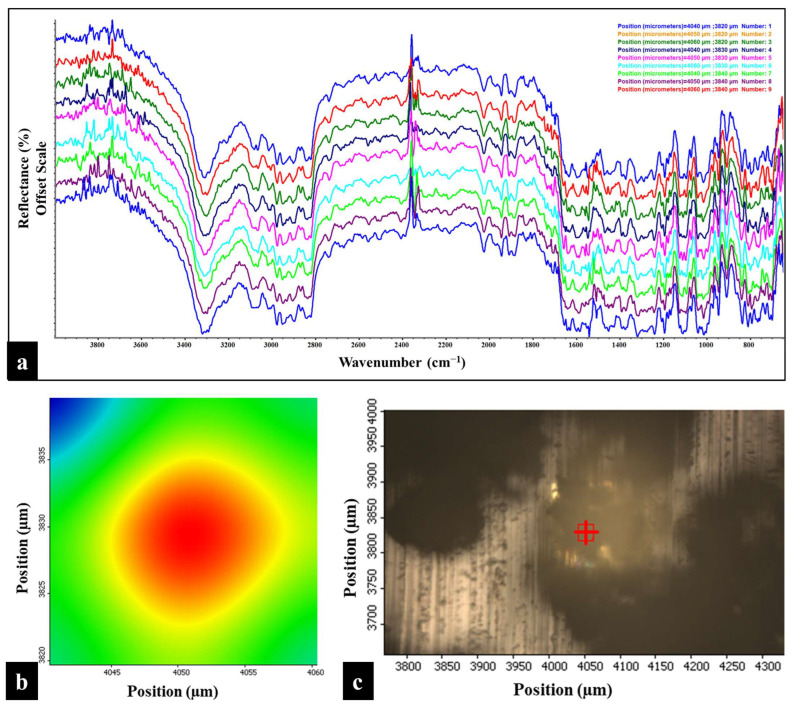
(**a**) FTIR map of BMS-202 powders showing the spectra obtained from nine distinct points on the sample. Y-axis of each overlay map is represented on an offset scale. (**b**) 2D area contour map of the highlighted red square region (as shown in (**c**)), displaying the position coordinates of each spectrum. (**c**) 2D area map displaying the sample surface under a 10× objective, with a highlighted red square indicating the specific region analyzed. Each spectrum in the overlay corresponds to one of the nine points within the highlighted red square and color-coded according to the spectrum obtained.

The ATR-FTIR chemical imaging map of BMS-202 powder, shown in Figure 9, was generated by analyzing the FTIR spectra at nine distinct points (position coordinates). The overlay of these spectra demonstrates the spatial uniformity of the powder bed, indicating chemical uniformity across the sample. The spectra, obtained from different physical locations represented by two-dimensional positional coordinates, show that the powder is uniform in chemical composition, with no significant impurities, as evidenced by the consistent overlay without deflections in reflectance.

### 3.10. Karl Fisher (KF) Coulometric Titration

As shown in Table 3, the residual water content of BMS-202 was found to be 2.76 ± 1.37% (*w*/*w*).

### 3.11. Gravimetric Vapor Sorption (GVS)

**Figure 10:**
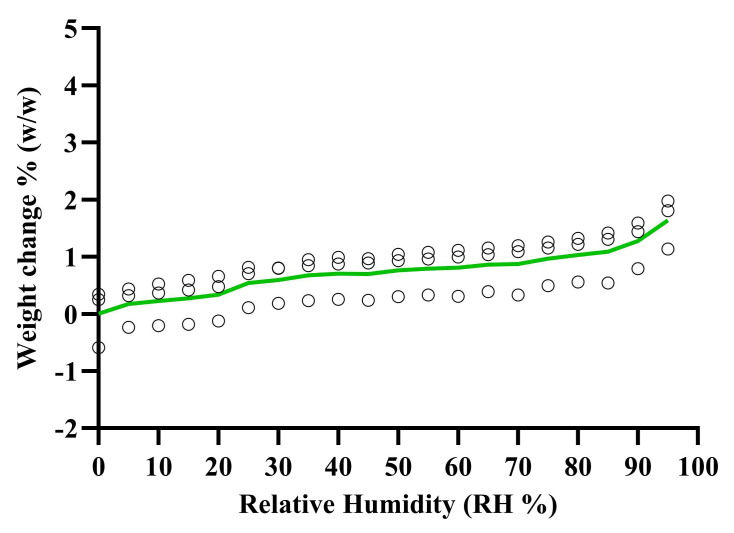
Gravimetric water vapor sorption isotherm of BMS-202 powder (weight change % vs. RH) at room temperature (25 °C). The green line represents the average of three individual experiments. Each circle represents the percent weight change when exposed to different levels of the humidity.

The vapor sorption isotherms in Figure 10 show the percentage weight change of BMS-202 powder when exposed to different levels of relative humidity (RH) at 25 °C. The results indicate minimal weight change as the RH levels increase. Specifically, at 55% RH, the weight change is 0.79 ± 0.40% (*w*/*w*), and at 95% RH, it is 1.64 ± 0.44% (*w*/*w*). Under accelerated stability conditions of 75% RH, the weight change is 0.97 ± 0.42% (*w*/*w*). These results demonstrate that even at high humidity levels (up to 95% RH), BMS-202 powder does not absorb significant moisture from the environment.

### 3.12. Dose-Dependent In Vitro Cell Viability on Skin Epithelial Cells

**Figure 11:**
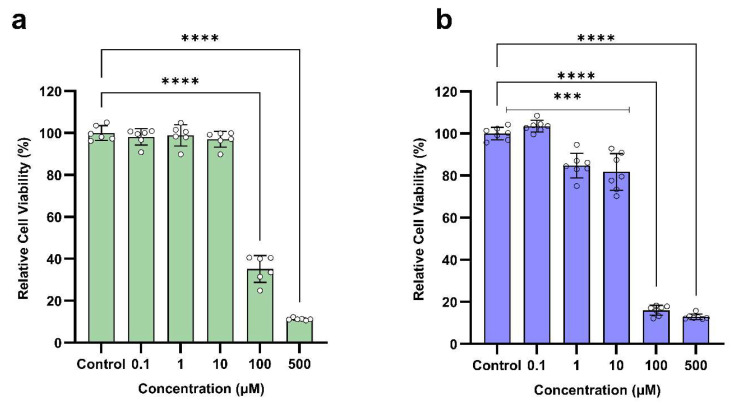
Dose-dependent in vitro cell viability of BMS-202 powder after 48 h of exposure on skin epithelial cells. (**a**) HaCaT (mean ± SD, n = 6), Control: 100.00 ± 3.54, 0.1 µM: 98.14 ± 3.88, 1 µM: 98.85 ± 5.07, 10 µM: 97.08 ± 3.81, 100 µM: 35.15 ± 6.37, and 500 µM; 11.25 ± 0.57 (**b**) NHEK (mean ± SD, n = 7), Control: 100.00 ± 2.98, 0.1 µM: 103.46 ± 2.73, 1 µM: 84.84 ± 5.84, 10 µM: 81.73 ± 8.74, 100 µM: 15.98 ± 2.35, and 500 µM: 12.87 ± 1.34. Each small circle represents a data point, and the vertical bars on each column indicate the standard deviation of the data. One-way ANOVA was used to compare different dose treatments with control (no treatment) using Dunnett’s multiple comparison test (α = 0.05, *** *p* < 0.001, and **** *p* < 0.0001).

The relative cell viability of different doses of BMS-202 after 48 h of exposure to two different skin epithelial cells (HaCaT and NHEK) is shown in Figure 11. The in vitro results indicate a dose-dependent response, with significant reductions in cell viability of both HaCaT and NHEK at higher concentrations of 100 µM and 500 µM. At lower concentrations ranging from 0.1 µM to 10 µM, the cell viability of HaCaT cells remains high (above 97%), indicating no toxicity. In this dose range, the cell viability of NHEK slightly decreases at 1 µM (84.84%) and 10 µM (81.73%), but these reductions are not as drastic, suggesting acceptable safety at these dose levels. However, at higher dose levels of 100 µM and 500 µM, there is a significant reduction in cell viability of both HaCaT and NHEK cells, indicating toxic effects. These findings suggest that BMS-202 exhibits cytotoxic effects on skin epithelial cells at higher doses, while lower doses are safe for application as a topical formulation.

### 3.13. In Vitro Transepithelial Electrical Resistance (TEER) with Skin Epithelial Cells at the Air–Liquid Interface (ALI)

**Figure 12:**
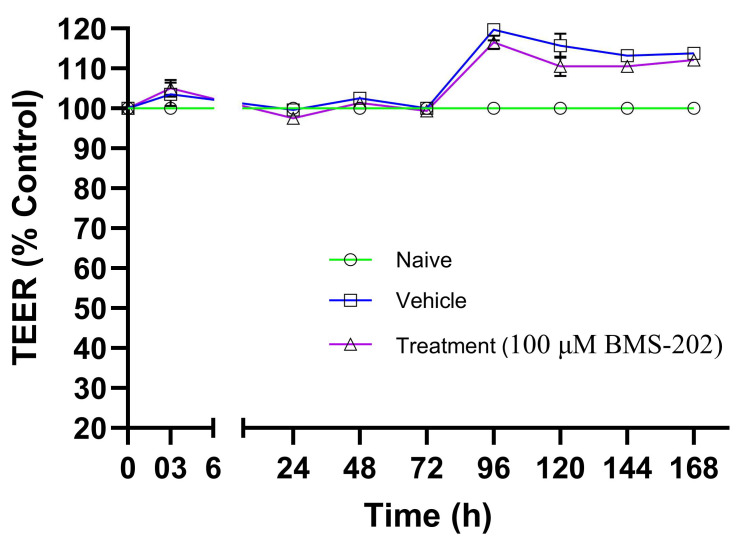
Transepithelial electric resistance (TEER) values (% of control) recorded at various time points using a STX4 open-ended electrode connected to an EVOM™ TEER measurement meter. Measurements were taken across HaCaT cells in Transwells^®^ under air–liquid interface (ALI) conditions after exposure to 100 µM BMS-202. TEER values were also recorded for naïve (non-treated) and vehicle-treated Transwells^®^. Each time point represents *n* = 4 replicates. Statistical analysis was conducted using a two-way ANOVA with Dunnett’s multiple comparison test (*p* < 0.05) to compare the means across different time points.

Figure 12 shows the TEER values, expressed as a percentage of control, recorded after 3 h and subsequently for 7 days post-treatment with 100 µM BMS-202 or vehicle, or under untreated (naïve) conditions. The results indicate that the TEER values remain stable across all tested conditions throughout the 7-day study period. The slight increase in TEER values observed after 3 days suggests a strengthening of barrier properties, likely due to normal cellular barrier reinforcement responses over time. Since there is no statistical difference in the TEER values between the vehicle or BMS-202-treated HaCaT cell layers at any time point, it can be concluded that neither the drug nor the vehicle used to solubilize the drug causes any transient decrease or increase in TEER values throughout the study period.

## 4. Discussion

In this study, we comprehensively characterized the physicochemical properties of BMS-202 and evaluated its effects on skin epithelial cells. To the best of our knowledge, this is the first report to detail the morphological, thermal, and spectroscopic properties of BMS-202, as well as its dose-dependent effects on cell viability and its impact on epithelial barrier integrity.

All analyses in this study were performed on a single batch of BMS-202 procured from Ambeed, Inc. (Arlington Heights, IL, USA). It is known that it is possible to have variation in manufacturing procedures between different vendors, which may lead to variations in purity, polymorphic forms, phase transition temperatures, solubility, and/or particle size. These variations can, in turn, affect performance properties such as dissolution and stability. The potential variability associated with different sources of active pharmaceutical ingredients (APIs) is well-documented in the literature, which is why general standard practice in pharmaceutical research is to maintain consistency in using one vendor source and to use the highest purity available, if possible. In addition, adhering to and implementing current Good Manufacturing Practices (cGMP) regulations and Quality by Design (QbD) standards, batch-to-batch variation is minimized. These measures are designed to ensure consistent product performance and regulatory compliance.

The predicted physicochemical properties of BMS-202 (Table 1) are generally favorable for topical delivery. Its moderate molecular weight and lipophilicity (logP between 4.37 and 5.10) suggest good potential for permeation through the lipid-rich environment of the skin′s outer layer, the stratum corneum [38,45]. The pKa of 8.08, indicating that the compound will remain largely un-ionized at physiological skin pH, further supports this, as un-ionized forms of drugs are better absorbed through the skin [38]. The tPSA value of 75.64 Å², well below the 140 Å² threshold often considered optimal for skin absorption, also suggests that BMS-202 could effectively penetrate the skin layers [38]. However, its low water solubility (negative logS value) must be considered when formulating a cream, as appropriate solubilizers or emulsifiers will be needed to ensure uniform distribution within the cream matrix. The data suggest that while BMS-202 is well suited for topical application, specific formulation strategies will be necessary to overcome its solubility limitations, ensuring effective delivery to the target site. According to Lipinski’s Rule of Five (molecular weight of 419.48 g/mol; log octanol–water partition coefficient of 4.11; tPSA of 75.64 Å²; 6 hydrogen bond acceptors, and two hydrogen bond donors), BMS-202 is also suitable for oral administration, but will pose challenges in ensuring solubility to get a favorable pharmacokinetic and pharmacodynamic profile for oral bioavailability [46]. The software-predicted melting point is notably higher than the value determined experimentally in this study using DSC. However, when utilizing the Quest Predict™ Melting Point Predictor tool (https://www.aatbio.com/tools/predictive-modeling/melting-point-predictor, last accessed on 23 September 2024), we found that the predicted value of 159.12 °C closely aligns with the experimentally determined melting point using DSC.

The SEM analysis (Figure 2) of BMS-202 powder particles revealed agglomerated particles with irregular shapes and rough surfaces. The agglomerated particles are likely formed through the interlocking of tabular, crystal-like structures. These particles also exhibited pores and cracks, forming a hollow structure composed of clustered micro-crystallites. The particle size analysis showed a wide size distribution, ranging from 8.31 to 92.61 µm, with an average geometric diameter of 30.91 ± 19.43 µm. Complementing these findings, the SEM-EDX spectroscopy (Figure 3) provided the elemental composition of the particles, confirming the presence of carbon (C), nitrogen (N), and oxygen (O) as the primary elements, which aligns with the compound′s molecular structure. The absence of trace impurities, especially heavy elements, corroborates the manufacturer′s stated purity of 98%. These combined observations suggest that BMS-202 possesses a consistent elemental profile and morphological characteristics suitable for its intended pharmaceutical applications.

The DSC thermogram of BMS-202 (Figure 4) demonstrated a clear melting point at 110.90 ± 0.54 °C, with an associated enthalpy of fusion, confirming its crystalline nature, the fact supported by many studies [47,48,49]. This finding was further corroborated by HSM (Figure 5), which visually displayed birefringence (an optical property observed in anisotropic materials, where different refractive indices appear along different optical axes) up to the melting point, indicative of a crystalline state [39]. The transition to an amorphous glassy state upon melting was also evident, aligning well with the DSC data. The presence of a high-temperature exothermic peak indicates that BMS-202 maintains stability up to 240 °C before undergoing substantial degradation. The crystalline nature of BMS-202 suggests that the compound is stable under a range of temperatures, which is beneficial for storage and handling. BMS-202 has demonstrated promising results in melanoma models by inhibiting PD-1/PD-L1 binding, which enhances the production of IFN-γ, a crucial cytokine that boosts the immune system′s ability to target cancer cells [20]. For topical formulations such as creams or gels, especially in the context of skin cancers like melanoma, employing strategies such as suitable solubilizers or stabilizers, permeation enhancers, and selecting appropriate formulation bases is essential to maintain the crystalline form and stability of BMS-202. From a formulation perspective, processing techniques like milling, granulation, or micronization should be carefully conducted at temperatures well below the compound′s melting point to prevent decomposition or phase transition. Additionally, topical patches providing controlled release and improved patient adherence could be considered as an effective delivery system for BMS-202 [50].

Raman and FTIR spectroscopies (Figure 6 and Figure 8) provided detailed insights into the molecular vibrations and structural composition of BMS-202. The sharp peaks observed in the Raman spectrum confirm the crystalline structure, with specific vibrational modes corresponding to functional groups such as N-H, C-H, C=C, C=N, and C-O bonds. These peaks indicate well-defined molecular packing, which supports the DSC and HSM findings of crystallinity. Additionally, Raman and FTIR chemical imaging maps (Figure 7 and Figure 9) confirmed the chemical uniformity of BMS-202 powder. The spectra obtained from multiple points on the sample showed consistent peak patterns, indicating uniform spatial distribution and absence of impurities. The uniformity in chemical composition suggests that BMS-202 is well suited for applications requiring consistent dosing and delivery, such as topical formulations. The uniformity ensures that each application delivers a predictable amount of active pharmaceutical ingredient, which is crucial for achieving reliable therapeutic outcomes. These findings provide a robust foundation for further formulation development, with considerations like selection of suitable bases, permeation enhancers, or other excipients necessary for the topical preparation of this molecule, ensuring stability and efficacy in its final formulation. The insights gained from these spectroscopic analyses not only confirm the suitability of BMS-202 for drug development but also guide the formulation strategies to maximize its therapeutic potential.

The residual water content (Table 3) of BMS-202 was found to be 2.76 ± 1.37% (*w*/*w*). While the United States Pharmacopeia (USP) and the International Council for Harmonization (ICH) guidelines do not provide a universal limit for residual moisture content in drug molecules for topical preparations, certain general considerations are important when preparing such formulations [51]. Firstly, it is essential to ensure that the moisture content does not lead to hydrolysis or degradation of the drug, as this could compromise the efficacy and safety of the formulation. Additionally, the residual moisture should not adversely affect the viscosity or texture of topical applications, which are critical for patient compliance and the effective application of the product. In formulations such as creams or gels that typically contain water, the residual water content must be accounted for to maintain the desired consistency and stability throughout the shelf life of the product. If transdermal patches are to be developed using BMS-202, the water content will play a crucial role in determining the adhesive properties and drug release kinetics of the patches. Moreover, it is important to ensure that the moisture content does not promote microbial growth, which would necessitate the use of preservatives [52]. The moisture level can also influence the type of packaging required to maintain the product′s integrity, ensuring that it remains stable and effective throughout its intended shelf life.

The vapor sorption isotherms (Figure 10) indicate minimal weight change as the RH levels increase. These results demonstrate that even at high humidity levels (up to 95% RH), BMS-202 powder does not absorb significant moisture from the environment. This low moisture uptake reduces the risk of environmental hydrolysis and moisture-related degradation, making BMS-202 chemically stable under varying humidity conditions. The non-hygroscopic nature of BMS-202 is likely due to its stable crystalline structure, which resists moisture uptake compared to amorphous forms with less ordered molecular structures [53]. From a topical formulation perspective, the low hygroscopicity of BMS-202 helps maintain the consistency and stability of the formulation. The minimal moisture absorption reduces the risk of microbial contamination, allowing for a more cost-effective choice of packaging materials [51]. Additionally, the stable nature of BMS-202 provides flexibility in selecting excipients based on the desired release profile and type of formulation, further enhancing its utility in topical drug delivery.

The dose-dependent cell viability assays (Figure 11) revealed that BMS-202 is cytotoxic to HaCaT and NHEK cells at higher concentrations (100 µM and 500 µM), while lower concentrations (0.1 µM to 10 µM) were found to be safe, particularly for HaCaT cells. This dose range provides a therapeutic window that could be leveraged for safe and effective topical formulations. HaCaT cells are immortalized keratinocytes derived from adult human skin that have undergone spontaneous transformation, allowing them to divide indefinitely in culture while maintaining characteristics of normal keratinocytes. On the other hand, NHEKs (normal human epidermal keratinocytes) are primary cells isolated from the epidermis of humans, typically through skin biopsies, and provide a physiologically relevant model for understanding the normal behavior of human keratinocytes. We conducted dose-ranging studies on both these cell line models to offer a balanced approach to the study. HaCaT cells provide a stable and reproducible model, as they can be cultured indefinitely, making them suitable for high-throughput screening. However, these cells have altered genetic and phenotypic characteristics compared to normal cells that may affect their response to drug treatments. Therefore, NHEKs provide physiological relevance as they more closely mimic normal human epidermal cells and can be useful for providing more clinically relevant data.

Comparative analysis of the data from both in vitro cell line models reveals the same safe (non-toxic) BMS-202 doses, ranging from 0.1 µM to 10 µM. Doses ranging from 100 µM to 500 µM were significantly toxic in both models. In terms of the implications of this study for drug discovery, especially formulation development, this therapeutic window (0.1 µM to 10 µM) is crucial for further preclinical studies and dosing strategies that maximize efficacy while minimizing toxicity. The consistent response in both cell models supports the hypothesis that the mechanism of action of BMS-202 works across different epidermal cell types. This could be very effective in skin-related cancers that involve PD-1/PD-L1 pathways.

A review of the existing literature provides valuable insights into the pharmacodynamics of BMS-202. The IC_50_ value, which indicates the concentration at which 50% inhibition of the PD-1/PD-L1 interaction is achieved, has been reported to be 18 nM, based on a homogeneous time-resolved fluorescence (HRTF) binding assay [54]. Additional studies suggest moderate IC_50_ values in the range of 113 nM to 146 nM [55,56], indicating that BMS-202 is a potent inhibitor within this interaction framework. Similarly, the EC_50_ value, representing the concentration required to restore 50% of T cell activity (measured in immune checkpoint blockade co-culture assays), varies between studies, with reported values ranging from 293 nM to 566 nM [56,57,58,59]. This variability likely stems from differences in experimental models or assay conditions. This suggests that BMS-202 has a broad therapeutic window, which could make it suitable for therapeutic applications where a balance between efficacy and toxicity is crucial.

While the effective dose ranges for small molecules vary according to the intended use, in oncology, the maximum tolerated dose (MTD) is often used as a benchmark [60]. However, this approach has limitations regarding targeted therapies, where an alternative approach called the biologically efficacious dose (BED), which balances safety and efficacy, is used [61]. The in vitro therapeutic safety window of BMS-202 (0.1 µM to 10 µM) suggests that the molecule has a relatively broad range that can be effective without causing significant toxicity, thereby allowing dosing flexibility. Future studies will involve dose optimization in preclinical models, demonstrating pharmacokinetics, studying combination therapies, and exploring therapeutic potential and limitations in other cancer models.

The TEER assay results (Figure 12) demonstrated that BMS-202 does not compromise the barrier integrity of HaCaT epithelial cells at the concentration tested (100 µM). The stable TEER values suggest that BMS-202 does not disrupt tight junctions or alter membrane permeability, which is essential for maintaining barrier function in topical applications [62]. The stable TEER values may also imply that the transport of BMS-202 across the epithelial cell layer occurs without affecting the tightness and integrity of the membrane.

Based on these results, it can also be inferred that BMS-202 is likely transported across the HaCaT epithelial cell layer primarily via passive diffusion or carrier-mediated transport, as these mechanisms do not compromise the epithelial barrier [63]. It is important to note that the same concentration of BMS-202 (100 µM) used in the dose-dependent cell viability study was found to be toxic to HaCaT cells. These results may seem contradictory; however, this discrepancy is due to the nature of the different experiments performed. The TEER assay measures membrane integrity and barrier tightness as a drug molecule passes across a membrane [64]. The drug molecule may utilize a cellular transport pathway that could result in a temporary or permanent disruption of these properties. In the TEER assay, drug molecules pass from the apical side to the basolateral side of the membrane, while in the cell viability assay, drug molecules remain in the well for the duration of the study [64]. The TEER values provide an indication of the temporal effects of the drug on barrier function early in the transport process, whereas intracellular accumulation of the drug in the cell viability assay can lead to toxicity. These differences explain why TEER values remain stable throughout the study, despite the same concentration of BMS-202 being toxic to cells in the cell viability experiment. Overall, these results suggest that BMS-202 does not disrupt tight junctions between cells, which are crucial for maintaining selective permeability, nor does it cause any adverse effects related to barrier disruption. The absorption of the drug across the epithelial layer likely occurs through passive diffusion, which is consistent and predictable, ensuring effective concentrations at the target site. Based on these findings, the formulation of a topical delivery system for BMS-202 would need to focus on using an appropriate vehicle, permeation enhancers, suitable pH, osmolarity, and skin-compatible excipients.

The comprehensive characterization of BMS-202 reveals its crystalline nature, chemical uniformity, and favorable interaction with skin epithelial cells at certain concentrations. These findings provide a solid foundation for further development of BMS-202 as a topical therapeutic agent, particularly in formulations requiring consistent dosing and stability. The data generated from this study also offer valuable insights for optimizing the formulation process, including the choice of excipients, permeation enhancers, and packaging materials. Future studies should focus on in vivo evaluations and clinical trials to further establish the safety and efficacy profile of BMS-202 for topical applications.

## 5. Conclusions

This study provides a comprehensive characterization of BMS-202, focusing on its physical properties, safety profile, and stability under varying conditions. DSC and HSM analyses confirmed that BMS-202 is a crystalline compound with a well-defined melting point and thermal behavior. Raman and FTIR spectroscopy, along with their microscopies, validated the chemical structure and uniformity of the powder, ensuring consistency in formulation. The residual moisture content and vapor sorption isotherms indicated that BMS-202 exhibits low hygroscopicity, which reduces the risk of moisture-related degradation and microbial contamination. This stability is advantageous for maintaining formulation consistency and effectiveness. In vitro studies on skin epithelial cell lines demonstrated a dose-dependent toxicity of BMS-202, with significant cytotoxic effects observed at concentrations of 100 µM and 500 µM. However, lower doses (0.1 µM to 10 µM) were well tolerated, supporting the safety of these concentrations for topical applications. TEER studies revealed that BMS-202 does not disrupt the tight junctions or barrier function of HaCaT cells, indicating that the compound can be transported across epithelial layers without compromising membrane integrity.

In conclusion, BMS-202′s favorable physical and chemical properties, along with its low moisture absorption and safety profile at therapeutic doses, make it a promising candidate for development into topical formulations. The findings will contribute to the formulation optimization of BMS-202, ensuring consistent therapeutic outcomes with minimal systemic exposure, maximizing its potential in treating skin cancers and other dermatological conditions.

## Figures and Tables

**Table 1:** Computational predictions of physicochemical parameters of BMS-202 (drawn using ChemDraw^®^ ver. 21.0.0 (ChemOffice, Cambridge, MA, USA)) and Molecular Modeling Pro Plus^®^ ver. 8.0.4 (Norgwyn Montgomery Software Inc., North Wales, PA, USA).

Property	ChemDraw^®^	Chem3D^®^	Molecular Modeling Pro^®^
Exact Mass	419.22 g/mol	419.220891806 g/mol	N/A
Mol Weight	419.53 g/mol	419.525 g/mol	419.4833 g/mol
Mol Formula	C_25_H_29_N_3_O_3_	C_25_H_29_N_3_O	C_25_H_29_N_3_O
LogP	4.37 Log Units	5.10126 Log Units	4.3251 Log Units
CLogP	4.40815 Log Units	4.40815 Log Units	4.41308 Log Units
LogS	–6.084 Log Units	–6.08447 Log Units	–5.72508 Log Units
pKa	8.080 Log Units	8.080 Log Units	N/A
tPSA	71.95 Å^2^	71.95 Å^2^	75.64 Å^2^
Melting Point	562.07 °C	N/A	N/A

N/A: not available.

**Table 2:** DSC thermal analysis data (*n* = 3, mean ± SD) of BMS-202 powder showing two primary thermal transitions.

Transition Type	Endothermic Peak	Exothermic Peak
Enthalpy (J/g)	84.41 ± 0.38	198.92 ± 9.85
Peak Temperature (°C)	110.90 ± 0.54	260.87 ± 2.02

**Table 3:** Residual water content of BMS-202 powder (*n* = 3) calculated using KF coulometric titration.

Samples	Water Content (% *w*/*w*)
BMS-202 *n* = 1	1.909
BMS-202 *n* = 2	2.028
BMS-202 *n* = 3	4.338
Average	2.758
Standard Deviation	±1.369

## Data Availability

The original contributions presented in the study are included in the article, further inquiries can be directed to the corresponding author.
